# Assessment of Anti-TB Drug Nonadherence and Associated Factors among TB Patients Attending TB Clinics in Arba Minch Governmental Health Institutions, Southern Ethiopia

**DOI:** 10.1155/2018/3705812

**Published:** 2018-02-18

**Authors:** Addisu Alemayehu Gube, Megbaru Debalkie, Kalid Seid, Kiberalem Bisete, Asfaw Mengesha, Abubeker Zeynu, Freselam Shimelis, Feleke Gebremeskel

**Affiliations:** ^1^Department of Public Health, College of Medicine and Health Sciences, Arba Minch University, P.O. Box 21, Arba Minch, Ethiopia; ^2^Department of Nursing, College of Medicine and Health Sciences, Arba Minch University, P.O. Box 21, Arba Minch, Ethiopia

## Abstract

**Background:**

Tuberculosis (TB) is an infectious disease caused by the bacillus* Mycobacterium tuberculosis*. Nonadherence to anti-TB treatment may result in the emergence of multidrug-resistant TB, prolonged infectiousness, and poor tuberculosis treatment outcomes. Ethiopia is one of the seven countries that reported lower rates of treatment success (84%). This study assessed anti-TB drug nonadherence and associated factors among TB patients in Arba Minch governmental health institutions.

**Methods:**

An institution based cross-sectional study design was conducted from April 15 to May 30, 2017. A systematic sampling technique was employed to select the study subjects. Data was collected using a semistructured questionnaire with Morisky Medication Adherence Scale-8 (MMAS-8) and was entered, cleaned, and analyzed in SPSS version 20.

**Results:**

The study included 271 TB patients with a response rate of 96.4%; 58.3% were males and 64.9% were Gamo by ethnicity. The overall nonadherence was 67 (24.7%) (CI = 20.0–30.4). Nonadherence was high if the patients experienced side effects (AOR = 13.332; 95% CI = 2.282–77.905), were far from the health facility (AOR = 21.830; 95% CI = 0.054–77.500), and experienced prolonged waiting time to get medical services (AOR = 14.260; 95% CI = 2.135–95.241).

**Conclusions:**

The proportion of TB patients that did not adhere to anti-TB drugs was high in Arba Minch governmental health institutions.

## 1. Introduction

Tuberculosis is an infectious disease caused by the bacillus* Mycobacterium tuberculosis*. It typically affects the lungs (pulmonary TB) but can also affect other sites (extrapulmonary TB). The disease is spread when people sick with pulmonary TB expel bacteria to the air, for example, by coughing. Overall, a relatively small proportion (5–15%) of the estimated 2-3 billion people infected with* Mycobacterium tuberculosis* will develop TB disease during their lifetime [1]. The prevalence of TB among close contacts of infectious patients can be about 2.5 times higher than in the general population [2]. If TB is detected early and fully treated, people with disease quickly become noninfectious and are eventually cured [3].

The WHO, in its global plan to stop TB, reports that poor treatment has resulted in the evolution of Mycobacterium* tuberculosis* strains that do not respond to treatment with the standard first-line combination of anti-TB medicines, resulting in the emergence of multidrug-resistant TB in almost every country of the world [4]. One of the greatest dilemmas and challenges facing most TB programs is a patient that does not complete TB treatment for one reason or another [5].

Poor adherence to treatment of chronic diseases including TB is a worldwide problem of striking magnitude [6]. However, patients with TB are expected to have adherence levels greater than 90% in order to facilitate cure. Failure of cure increases the risk of development of drug-resistant strains and spread of TB in the community, and this in turn increases morbidity and mortality [7, 8]. TB in 2015 was one of the top 10 causes of death worldwide. The best estimates are that there were 1.4 million TB deaths in 2015 and an additional 0.4 million deaths resulting from TB disease among HIV-positive people [1]. According to recent estimates, Ethiopia stands 7th in the list of high TB burden countries. In Ethiopia, TB is the leading cause of morbidity, the third cause of hospital admissions, and the second cause of death. The estimated TB incidence of Ethiopia was 261/100,000 inhabitants in 2011. The lifetime risk of developing TB in Ethiopia is estimated to be 50–60% for HIV-infected people and only 10% for HIV-negative counterparts [9, 10]. In many countries, globally, the adoption of Directly Observed Treatment (DOT) has been associated with reduced rate of treatment failure, relapse, and drug resistance. However, its impact on reducing TB incidence has been limited by noncompliance to DOT, which occurs when patients do not turn up for treatment at the health facility or community DOT point [11].

Despite the implementation of an internationally recommended strategy (DOT) in almost all parts of WHO regions and many national and international efforts exerted against TB prevention and control, still the patients fail to complete their treatment to be declared “cured” or “completed the treatment” [12–14]. Current WHO reports show that a considerable number of TB cases failed after several treatments; many relapsed after completion of the treatment, many had to undergo retreatment after completion of treatment, and many developed MDR-TB among retreatment cases (20%) throughout the world [15]. For this, most probably, treatment nonadherence and loss to followup are the main responsible factors [16]. Nonadherence to anti-TB treatment may result in the emergence of multidrug-resistant TB (MDR-TB), prolonged infectiousness, and poor TB treatment outcomes [17, 18]. In sub-Saharan Africa, there is a high rate of loss to followup of TB patients that ranged from 11.3% to 29.6% [19]. Ethiopia is one of the seven countries that reported lower rates of treatment success (84%) [9].

In Ethiopia, even though TB drugs are given free of charge, TB continues to be a major health problem and cause of death. The Ethiopian national program for TB control recommends DOT as the main strategy for disease control, but its utilization differs due to local health institutions' capacities to guarantee patient supervision. Hence, this study will assess the level of nonadherence to anti-TB therapy and associated factors among TB patients in Arba Minch, Ethiopia.

## 2. Methods and Materials

### 2.1. Study Area and Period

The study was conducted in Arba Minch town, Gamo Gofa Zone SNNPR, Ethiopia. The town is located at 505 km from Addis Ababa and 275 km from Hawassa, capital city of Southern Ethiopia. The town is 30° 56′ north and 37° 44′ west, and it is located to the west of Lake Abaya. It covers 514 km^2^ and is generally located at the altitude of 1200 through 1400 meters above sea level. Based on the 2007 Ethiopian national population and housing census, the population of the town is projected to be about 86,405. The town has three public facilities: two health centers and one general hospital. Arba Minch General Hospital was established and started its full function in 1961 EC (Ethiopian Calendar). The hospital and the health centers are now providing several health services including TB treatment program for the community. The study was conducted from April 15 to May 30, 2017.

### 2.2. Study Population

The study population included all TB patients on anti-TB medication at least for a month at tuberculosis followup clinics in Arba Minch governmental health institutions.

### 2.3. Inclusion Criteria

All TB patients that were at least 15 years of age, regardless of the site or the smear status of their TB, and have taken anti-TB medication at least for a month were included.

### 2.4. Sample Size and Sampling Techniques

The sample size was calculated based on a single population proportion formula using the following assumptions: *P* = 21% [20], with 95% confidence level and 5% level of precision. And by adding 10% for nonresponse rate, the final sample size was 281. In order to draw the sample, average flow of TB patients in each health institution per day was taken as a reference to estimate the client load. Based on the information, proportionate allocation to size was made in each institution. Therefore, 52% (146) of the sample was drawn from Arba Minch General Hospital, 32% (90) from Sikella Health Center, and the rest (16%, 45) from Shecha Health Center. Since TB patients receive the same TB clinic service. Systematic random sampling was used, and by dividing the total 436 patients to 281 patients, every 2nd TB patient was selected. The first study subject was determined randomly.

### 2.5. Data Collection Instrument and Procedures

Data was collected using a pretested and semistructured questionnaire. It was in English and was translated to Amharic and then back to English to check for consistency and completeness. Then, it was collected through a face-to-face exit interview.

### 2.6. Operational Definitions

Nonadherence to anti-TB drugs means an individual whose score > 2 points in the Morisky Medication Adherence Scale-8.

MMAS-8 consists of eight items, with a scoring scheme of “yes” = 0 and “no” = 1 for the first seven items, but for the last item, a five-point Likert scale response will be used with options of “never,” “once in a while,” “sometimes,” “usually,” and “always.” In this Likert scale, values ranging from 0 to 1 were given at a specified interval of 0.25 with “0” given for “never” and “1” given for “always.” The degree of adherence was determined according to the score resulting from the sum of all the correct answers to a maximum score of 8. For the purpose of data analysis, the three original categories of adherence were recategorized into two. Accordingly, high and medium adherence were reassigned as adherent with a score of less than or equal to 2 and low adherence will be regarded as nonadherent with a score of greater than 2.

### 2.7. Data Processing and Analysis

After checking for completeness and consistency, data was entered into SPSS (IBM 20) for descriptive and inferential analysis. Binary logistic regression was used to determine the dependent variable on the basis of continuous and/or categorical independent variables, and factors with *P* value ≤ 0.25 in bivariate analysis were candidates for multivariate analysis and factors with *P* < 0.05 in the final model were statistically significant. The degree of association between dependent and independent variables was assessed using AOR at 95% CI.

### 2.8. Ethical Consideration

Ethical clearance was obtained from the ethical review committee of the College of Medicine and Health Sciences, Arba Minch University. A formal letter was given to each of the public health institutions in Arba Minch town. In addition, informed consent was obtained from study participants to confirm their willingness for participation after explaining the objective of the study. And the respondents were notified that they have the right to withdraw at any point of the interview.

## 3. Results

### 3.1. Sociodemographic Characteristics of the Study Participants

From 281 selected participants, 271 were involved in the study with a response rate of 96.4%. Of these, 142 (52.4%) were aged 25–34, with a mean age of 32.19 (±11.291 SD) years. Of 271, 158 (58.3%) were males and 170 (62.7%) were protestants. 190 (70.1%) of the participants were married and 176 (64.9%) were Gamo by ethnicity. 82 (30.3%) of the study participants attended grades 7–12, 183 (67.5%) were self-employed, and 169 (62.4%) got more than 501 birr as a monthly income (Table 1).

### 3.2. Behavioral Risk Factors of Study Participants

249 (91.9%) of the study participants had no smoking habit and 227 (83.8%) never drank alcohol before. 15 (5.5%) of the study participants had no treatment supporter (Table 2).

### 3.3. Healthcare System Related Characteristics of the Study Participants

150 (55.3%) stated that their preferable TB clinic opening time was from 2:00 AM to 6:00 PM local time, and for 225 (83.0%), the waiting time at the health facility was <1 hour. 56 (20.7%) of the participants reported that it took them more than 5 kms and 5 birr to reach the nearby TB clinic.

44 (16.2%) of the study participants stated that no one supervised them while taking their TB medication. 208 (76.8%) reported that the health workers were friendly to them.

259 (95.6%) participants had good knowledge about TB but 12 (4.4%) participants did not know the symptoms of TB at all. 46 (17.0%) of the participants reported that they stopped taking their anti-TB medication when they felt better. 249 (91.9%) disclosed their illness to their relatives, and all those who did not disclose had fear of stigma and discrimination (Table 3).

### 3.4. Disease and Medicine Related Factors

30 (11.1%) of the TB patients reported some kind of anti-TB medication adverse effects: of these, 10 (33.4%), 9 (30%), 7 (23.3%), and 4 (13.3%) participants complained of minor adverse effects, that is, vomiting and diarrhea, numbness of feet and hands, headache and dizziness, and skin rash, respectively. 257 (94.8%) of the participants reported that they felt better in less than 2 months' time after starting anti-TB medication.

229 (84.5%) of the TB patients were screened for HIV and 6 (2.2%) of them were positive. 14 (5.2%) of the TB patients have taken drugs other than anti-TB medication: of these, 6 (42.8%) for HIV, 2 (14.3%) for pneumonia, 1 (7.1%) for STI, and 5 (35.8%) for fungal diseases (Table 4).

### 3.5. Prevalence of Nonadherence to Anti-TB Medication

**Figure 1:**
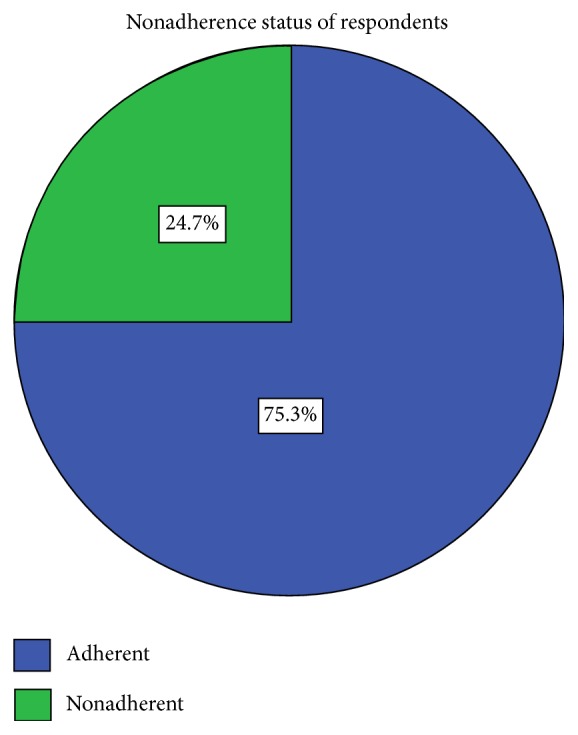
Adherence status of the TB patients that attended TB clinics at governmental health institutions of Arba Minch town, Southern Ethiopia, 2017 (*N* = 271).

The overall calculated nonadherence in this study was 67 (24.7%) with a confidence interval (CI) of 20.0–30.4 (Figure 1).

### 3.6. Factors Associated with Nonadherence to Anti-TB Treatment

In bivariate analysis, sex, educational status, waiting time at health facility, distance to health facility, side effects of the drugs, smoking, alcohol use, treatment supporter, and monthly income were significantly associated with nonadherence to anti-TB medication. But in multivariate analysis, patients who experienced drug side effects, those who were far from the health facility, and those that experienced a prolonged waiting time to get medical services remained significantly and independently associated with anti-TB medication nonadherence. Those patients that experienced side effects were thirteen times more likely (AOR = 13.332; 95% CI: 2.282–77.905) to be nonadherent than their counterparts. In addition, patients that came from a far distance to the health facility to get medical services (AOR = 21.830; 95% CI: 0.0–554–77.500) and those who experienced a prolonged waiting time at the health facility to get medical services (AOR = 14.260; 95% CI: 2.135–95.241) were also at a higher risk of nonadherence than those who were near the health facility and those that waited for a short period of time, respectively (Table 5).

## 4. Discussion

This study assessed anti-TB drug nonadherence and the associated factors among TB patients in Arba Minch governmental health institutions. Even if it is recommended that every TB patient should adhere to anti-TB medication by following DOT strategy [1], the findings of this study showed that 24.7% of TB patients did not adhere to anti-TB drugs. This finding is almost in line with the finding of the study conducted in a tertiary health institution in Southeast Nigeria and in South Ethiopia where nonadherence for anti-TB drugs was 24.2% and 24.5%, respectively [21, 22]. In this study, the proportion of TB patients that do not adhere to anti-TB drugs is higher compared to the findings of the study conducted in Southwest Ethiopia and Northwest Ethiopia where the nonadherence was 20.8% and 10%, respectively [20, 23]. This discrepancy could be, in the first case, because the study was conducted in three big towns in North Ethiopia and, in the second case, could be because the study finding only represents the result from a single hospital and only few patients. In the current study, the number of TB patients that did not adhere to anti-TB drugs is relatively smaller than that found by a study conducted in Hadiya Zone, Southern Ethiopia, where nonadherence was 30% [24]. This might be attributable to the fact that the finding of the study conducted in Hadiya Zone represents TB patients both in higher health facilities and in rural health facilities, whereas our study was only restricted to patients from health facilities located in an urban setting, which is Arba Minch town.

In this study, one reason for nonadherence of anti-TB drugs is the waiting time at the health facility. While only 10.2% of TB patients waiting for less than 1 hour did not adhere to anti-TB drugs, 95.7% of TB patients waiting for 1-2 hours did not adhere. This is similar to the finding of the study conducted in Hadiya Zone, Southern Ethiopia, where the probability of complying with DOT is 2.5 times higher for TB patients waiting for less than 30 minutes compared to their counterparts [24].

The other important reason for anti-TB nonadherence among TB patients is distance to the health facility. While only 8.4% of TB patients 0–5 kms away from health facility did not adhere to anti-TB drugs, 87.5% of TB patients greater than 5 kms away from the health facility did not adhere. Similarly, the study conducted in South Ethiopia has depicted that the likelihood of not adhering to anti-TB drugs for TB patients faraway from DOT center is 5.7 times higher than TB patients nearer to the center [22].

Side effects of the drugs are also an important reason for anti-TB drug nonadherence among TB patients. While the likelihood of not adhering to anti-TB drugs among TB patients with side effects of the drugs is 90%, only 16.6% of TB patients without the side effects of the drugs did not adhere to anti-TB drugs. This finding is in line with the finding of the study carried out in Tigray, Northern Ethiopia, where the probability of adhering to anti-TB drugs among TB patients without drugs side effects was 3 times higher than in those with drugs side effects [25]. Similarly, in a study carried out in Alamata District, Northeast Ethiopia, one of the main reasons for nonadherence was the presence of drugs side effects among TB patients [26].

In this study, some variables which were significant during bivariate analysis were not found to be statistically significant during multivariate analysis and were not independent determinants of anti-TB drug nonadherence among TB patients. One of such variables was sex, which in this study has no effect on anti-TB drug nonadherence among TB patients. Similarly, studies conducted in Southeast Nigeria and Plateau State, Nigeria, have shown that sex has no significant effect on anti-TB drug nonadherence among TB patients [21, 27]. On the contrary, the study conducted in Argentina has shown that difference in sex has an effect on anti-TB drug nonadherence among TB patients [28]. The other variable with no effect on anti-TB drug nonadherence in this study was educational status. In the same manner, studies carried out in South Ethiopia and Plateau State, Nigeria, have indicated that the educational status of TB patients has no effect on anti-TB drug nonadherence [22, 27]. But studies conducted in Southwest Ethiopia and Southeast Nigeria have shown that educational status has a significant effect on anti-TB drug nonadherence among TB patients [20, 21]. Similarly, having a treatment supporter in this study has no effect on anti-TB drug nonadherence, which is also in line with the finding of the study conducted in South Ethiopia [22]. The other variable that does not affect anti-TB drug nonadherence status in this study was alcohol use. In the same way, studies carried out in South Ethiopia and Plateau State, Nigeria, have revealed that alcohol use has no significant effect on anti-TB drug nonadherence [22, 27]. Cigarette smoking was another variable in this study that does not affect anti-TB drug nonadherence status, but the study conducted in Plateau State, Nigeria, has shown that cigarette smoking is significantly associated with anti-TB drug nonadherence among TB patients [27]. The last variable that is not associated with anti-TB drug adherence status among TB patients in this study was monthly income of TB patients, which is contrary to the finding of the study carried out in Southeast Nigeria where the average monthly income has significantly affected anti-TB drug adherence status of TB patients [21].

## 5. Conclusions

In general, this study revealed that the level of nonadherence to anti-TB drugs among TB patients in Arba Minch governmental health institutions is high. The waiting time at the health facility, the distance to the health facility, and the side effects of the drugs were significant determinants of nonadherence to anti-TB drugs. The other variables such as sex, educational status, smoking, alcohol use, treatment supporter, and monthly income were only significantly associated with nonadherence to anti-TB medication during bivariate analysis. And they were not found to be statistically significant during multivariate analysis.

## Figures and Tables

**Table 1:** Sociodemographic characteristics of the TB patients that attended TB clinics at governmental health institutions of Arba Minch town, Southern Ethiopia, 2017 (*N* = 271).

Variable	Frequency	Percent
*Age*		
15–24	65	24
25–34	142	52.4
35–44	30	11
>=45	34	12.6
*Sex*		
Male	158	58.3
Female	113	41.7
*Marital status*		
Married	190	70.1
Single	50	18.5
Divorced	21	7.7
Widowed	10	3.7
*Religion*		
Protestant	170	62.7
Orthodox	90	33.2
Muslim	11	4.1
*Ethnicity*		
Gamo	176	64.9
Wolaita	80	29.5
Gofa	15	5.5
*Educational status*		
Cannot read and write	25	9.2
Can read and write	62	22.9
Grades 1–6	75	27.7
Grades 7–12	82	30.3
Diploma and above	27	10.0
*Occupational status*		
Employee	57	21
Self-employed	183	67.5
No job	31	11.4
*Monthly income*		
<501 birr	102	37.6
>=501 birr	169	62.4

**Table 2:** Behavioral characteristics of the TB patients that attended TB clinics at governmental health institutions of Arba Minch town, Southern Ethiopia, 2017 (*N* = 271).

Variable	Frequency	Percent
*Smoking*		
Yes	22	8.1
No	249	91.9
*Alcohol*		
Yes	44	16.2
No	227	83.8
*Treatment supporter*		
Yes	256	94.5
No	15	5.5

**Table 3:** Healthcare system related characteristics of the TB patients that attended TB clinics at governmental health institutions of Arba Minch town, Southern Ethiopia, 2017 (*N* = 271).

Variable	Frequency	Percent
*Preferable time for TB clinic*		
2:00–6:00 AM	150	55.3
8:00–11:00 AM	120	44.3
After 2 PM	1	0.4
*Waiting time at health facility*		
<1 hr	225	83.0
1-2 hr	46	17.0
*Distance to health facility*		
0–5 km	215	79.3
>5 km	56	20.7
*Transport cost*		
0–5 birr	215	79.3
>5 birr	56	20.7
*Supervision*		
None	44	16.2
Family member	17	6.3
Health worker	210	77.5
*Relationship with health worker*		
Very friendly	22	8.1
Friendly	208	76.8
Indifferent	32	11.8
Unfriendly	9	3.3
*Knowledge on symptoms of TB*		
All	144	53.2
Some	115	42.4
Not knowing	12	4.4
*Time to stop TB medication*		
6–24 months	132	48.7
8–24 months	93	34.3
When feeling better	46	17.0
*TB status disclosure*		
Yes	249	91.9
No	22	8.1
*Reason for not disclosing*		
Fear of stigma and discrimination	22	100

**Table 4:** Disease and medicine related characteristics of the TB patients that attended TB clinics at governmental health institutions of Arba Minch town, Southern Ethiopia, 2017 (*N* = 271).

Variable	Frequency	Percent
*Experience of side effects*		
Yes	30	11.1
No	241	88.9
*Type of side effects*		
Vomiting and diarrhea	10	33.4
Headache and dizziness	7	23.4
Skin rash	4	13.3
Numbness of feet and hands	9	30
*Duration to feel better*		
<2 months	257	94.8
2–4 months	9	3.3
5-6 months	5	1.8
*Missing of anti-TB medication*		
Yes	69	25.5
No	202	74.5
*Reason for missing anti-TB medication*		
Forgetfulness	15	21.7
Vomiting and diarrhea	4	5.9
Cost of transport	5	7.2
Health professional attitude	3	4.3
Stigma and discrimination	7	10.1
Feeling better	35	50.8
*HIV status*		
Positive	6	2.2
Negative	223	82.3
Not tested	42	15.5
*Taking drugs other than anti-Tb medication*		
Yes	14	5.2
No	257	94.8
*Reason of taking another drug*		
HIV/AIDS	6	42.8
Pneumonia	2	14.3
STI	1	7.1
Fungal disease	5	35.8

**Table 5:** Factors associated with nonadherence of anti-TB drug treatment among TB patients that attended TB clinics at governmental health institutions of Arba Minch town, Southern Ethiopia (*N* = 271).

Variables	Nonadherence		Crude OR	Adjusted OR
	Yes	No		
*Sex*				
Male	50 (31.6%)	108 (68.4%)	1	1
Female	17 (15.0%)	96 (85.0%)	0.383 (0.207, 0.708)	3.023 (0.071,11.335)
*Educational status*				
Cannot read and write	15 (60.0%)	10 (40.0%)	5.250 (1.566, 17.601)	1.923 (0.023,8.013)
Can read and write	13 (21.0%)	49 (79.0%)	0.929 (0.311, 2.773)	0.745 (0.154,3.257)
Grades 1–6	19 (25.3%)	56 (74.7%)	1.187 (0.417, 3.380)	0.247 (0.099,6.227)
Grades 7–12	14 (17.1%)	68 (82.9%)	0.721 (0.246, 2.110)	2.016 (0.085,11.663)
Diploma and above	6 (22.2%)	21 (77.8%)	1	1
*Waiting time at health facility*				
<1 hour	23 (10.2%)	202 (89.8%)	1	1
1-2 hours	44 (95.7%)	2 (4.3%)	193.217 (43.929, 849.847)	14.260 (2.135,95.241)^**∗**^
*Distance to health facility*				
0–5 km	18 (8.4%)	197 (91.6%)	1	1
>5 km	49 (87.5%)	7 (12.5%)	76.611 (30.306, 193.669)	21.830 (5.278,90.284)^**∗**^
*Side effects of the drugs*				
Yes	27 (90%)	3 (10%)	45.000 (13.020, 155.528)	13.332 (2.282,77.905)^**∗**^
No	40 (16.6%)	201 (83.4%)	1	1
*Smoking*				
Yes	20 (90.9%)	2 (9.1%)	42.979 (9.708, 190.282)	0.903 (0.080, 10.200)
No	47 (18.9%)	202 (81.1%)	1	1
*Alcohol*				
Yes	43 (97.7%)	1 (2.3%)	363.708 (47.899, 2761.726)	2.665 (0.897, 9.776)
No	24 (10.6%)	203 (89.4%)	1	1
*Treatment supporter*				
Yes	53 (20.7%)	203 (79.3%)	1	1
No	14 (93.3%)	1 (6.7%)	53.623 (6.895, 417.018)	2.044 (0.054, 77.500)
*Monthly income*				
<501 birr	51 (50%)	51 (50%)	9.562 (5.018, 18.223)	2.267 (0.795, 6.468)
>=501 birr	16 (9.5%)	153 (90.5%)	1	1

^*∗*^Significant association.
